# Thiamin deficiency in children with chronic kidney disease on peritoneal dialysis and its association with dialysis duration and transport peritoneal membrane status

**DOI:** 10.1007/s00467-025-06847-6

**Published:** 2025-06-23

**Authors:** Wipawee Suwanboriboon, Thanaporn Chaiyapuk, Intraparch Tinnabut, Gornmigar Sanpawitayakul, Chatchawan Srisawat, Sarawut Junnu, Sompong Liammongkolkul, Kwanjai Chotipanang, Hathaichanok Rukprayoon, Phakwan Laohathai, Narumon Densupsoontorn

**Affiliations:** 1 Division of Nutrition, Department of Pediatrics, Faculty of Medicine Siriraj Hospital, Mahidol University, Bangkok, 10700 Thailand; 2 Division of Nephrology, Department of Pediatrics, Faculty of Medicine Siriraj Hospital, Mahidol University, Bangkok, 10700 Thailand; 3 Division of Ambulatory Pediatrics, Department of Pediatrics, Faculty of Medicine Siriraj Hospital, Mahidol University, Bangkok, 10700 Thailand; 4 Department of Biochemistry, Faculty of Medicine Siriraj Hospital, Mahidol University, Bangkok, 10700 Thailand

**Keywords:** Chronic kidney disease, Erythrocyte transketolase activity, High transporter, Pediatric patients, Peritoneal dialysis, Thiamin deficiency

## Abstract

**Background:**

Patients with chronic kidney disease (CKD) stage 5D receiving peritoneal dialysis (PD) are at risk for thiamin deficiency (TD). This study compared the proportion of TD in pediatric CKD patients undergoing PD with that in healthy controls and evaluated the associations of various factors with TD in CKD patients.

**Methods:**

Thirty-two patients with CKD stage 5D and 34 healthy children were recruited. The participants reported their consumption of foods containing antithiamin factors and completed a 3-day food record to assess their intake of thiamin, energy, and macronutrients. The medical records of the CKD group were reviewed. Thiamin status was assessed via an erythrocyte transketolase activity assay, where the thiamin pyrophosphate effect was determined.

**Results:**

Thirteen percent of participants in the CKD group had TD, whereas 29% of the healthy controls did (*p* = 0.093). The CKD group had significantly greater total thiamin intake per 1,000 kcal of energy due to thiamin supplementation (2.14 [1.83, 2.99] vs. 0.87 [0.59, 1.14] mg/1,000 kcal; *p* < 0.001), despite inadequate dietary thiamin intake. A longer PD duration (in months) and a high-transport peritoneal membrane status were significantly associated with poorer thiamin status (β = + 0.59, *p* < 0.001, and β = + 0.38, *p* = 0.013, respectively). In contrast, greater total thiamin intake was correlated with improved thiamin status (β = -0.35, *p* = 0.022).

**Conclusions:**

Thiamin deficiency was observed in 13% of pediatric CKD patients on PD and 29% of healthy controls. In CKD patients, TD was associated with longer PD duration (in months), high-transport peritoneal membrane status, and low total thiamin intake.

**Graphical abstract:**

**Figure:**
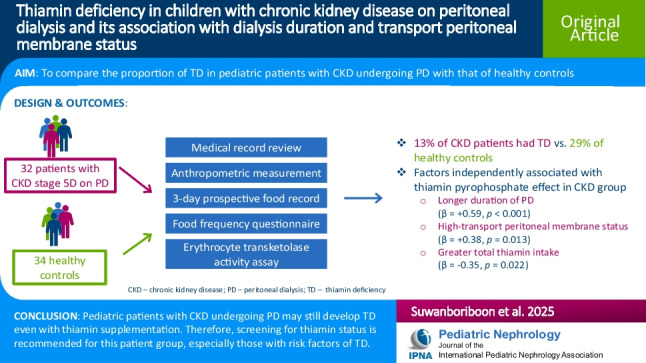
A higher resolution version of the Graphical abstract is available as Supplementary Information

**Supplementary Information:**

The online version contains supplementary material available at 10.1007/s00467-025-06847-6.

## Introduction

Chronic kidney disease (CKD) in children has heterogeneous causes. In many cases, CKD progresses to kidney failure, requiring kidney replacement therapy through either hemodialysis or peritoneal dialysis (PD) while the patient awaits kidney transplantation [1]. Children with CKD should aim to meet 100% of the estimated energy requirements for their chronological age, with adjustments in energy intake based on nutritional status and weight changes. However, many children experience poor appetite due to medications, as well as hormonal and cytokine changes associated with the disease [2]. Consequently, the intake of macronutrients and micronutrients may be inadequate [3, 4]. Additionally, factors such as nutrient loss during dialysis and disruptions in nutrient absorption, excretion, or metabolism can affect micronutrient levels in the body [2].

Thiamin is an indispensable water-soluble vitamin involved in several decarboxylation reactions. Thiamin deficiency (TD) can lead to two clinical manifestations: dry beriberi and wet beriberi. Dry beriberi affects the nervous system, resulting in peripheral neuropathy, paresthesia, muscle weakness, and brisk tendon reflexes. On the other hand, wet beriberi impacts the cardiovascular system, causing lactic acidosis, respiratory distress, and congestive heart failure. Daily thiamin requirements are typically met through diet, particularly from meats (especially pork), whole grains, legumes, sunflower seeds, and vegetables. However, certain foods contain antithiamin factors. Raw fish and shellfish, for example, have heat-labile thiaminases, whereas coffee, tea, and certain vegetables contain heat-stable thiamin antagonists [5–7].

Owing to its small molecular size, a substantial amount of thiamin can be lost during hemodialysis. Although thiamin loss through PD is generally considered minimal [2], a study in adults [8] reported that the amount of thiamin lost in peritoneal effluent over 24 h can exceed the dietary reference intake (DRI) requirement. Additionally, Warady et al. [9] found that 2 out of 5 infants with CKD who were undergoing PD had TD despite receiving a daily multivitamin supplement containing 0.6 mg of thiamin. The risk of TD among children with CKD is acknowledged by the Kidney Disease Outcomes Quality Initiative, which suggests thiamin supplementation for those unable to meet dietary requirements according to the DRI [2]. The primary aim of this study was to compare the proportion of TD in pediatric patients with CKD stage 5D who were undergoing PD with that in healthy controls. The associations between various factors and TD within the CKD group were also evaluated.

## Methods

### Participants

This cross-sectional study was conducted at the Faculty of Medicine Siriraj Hospital, Mahidol University, Bangkok, Thailand, from July 2021 to May 2024. The study protocol was approved by the Siriraj Institutional Review Board (approval number Si 552/2021). Informed assent was obtained from participants older than 7 years of age, and informed consent was obtained from each parent or legally authorized guardian. The inclusion and exclusion criteria were defined separately for the CKD patient group and the healthy control group. The inclusion criteria for the CKD group were children aged 1 to < 18 years with a diagnosis of CKD stage 5D who had been undergoing PD and were free from peritonitis for at least 1 month. The exclusion criteria comprised a history of small intestinal resection or bariatric surgery, which could increase the risk of thiamin malabsorption; comorbidities of red blood cell abnormalities, such as thalassemia, abnormal hemoglobin typing, and G-6-PD deficiency; and the use of any probiotic supplements containing thiamin-producing bacterial strains. For the healthy control group, participants were recruited from the Well Child Clinic of Siriraj Hospital and a nearby secondary school. Healthy controls were frequency-matched to the CKD group by sex and by age group, categorized as (1) < 11 years, (2) 11–15 years, and (3) > 15 years. The inclusion criteria were children aged 6 months to < 18 years with normal anthropometric measurements, indicated by World Health Organization (WHO) weight-for-age z-scores between −2 and + 2 and height-for-age z-scores between −2 and + 2 [10, 11]. The exclusion criteria consisted of any chronic underlying disease; history of small intestinal resection or bariatric surgery, which could increase the risk of thiamin malabsorption; severe anemia with hemoglobin < 7 g/dL or abnormalities of red blood cells, such as thalassemia, abnormal hemoglobin typing, and G-6-PD deficiency; use of any probiotic supplement containing thiamin-producing bacterial strains; use of thiamin-containing vitamin supplements; and age-inappropriate dietary practices, such as food restrictions, vegetarianism, or intermittent fasting.

### Sample size calculation

Although Warady et al. [9] reported that 40% of infants with CKD stage 5D undergoing PD had TD, we surmised that the prevalence of TD was less than 40%. Thus, we estimated the prevalence of TD in this group of patients to be 32% (20% less than the results reported by Warady et al. [9]). For the healthy pediatric population, Pongpaew [12] found that the prevalence of TD among Thai children of all ages was 5%. To compare the two independent proportions with an alpha of 0.05, a beta of 0.2, and a ratio of 1, a sample size of 32 participants was needed for each group.

### Participant characteristics and anthropometric evaluation

After written informed consent and assent were obtained, the anthropometric measurements of the participants from both groups were evaluated. Based on the WHO Child Growth Standards 2006 [10] and the WHO Growth Reference 2007 for ages 5 to 19 years [11], the weight-for-height z-score, BMI-for-age z-score, and height-for-age z-score were calculated for each participant. Wasting was defined as a weight-for-height z-score of less than −2 for children aged 5 years or younger, whereas thinness was defined as a BMI-for-age z-score of less than −2 for children older than 5 years of age. Stunting was indicated by a height-for-age z-score of less than −2 for children of all ages [13].

For participants in the CKD group, information regarding the causes of CKD, modes of PD, PD initiation date, 24-h urine volume, 24-h PD ultrafiltration volume, PD adequacy (Kt/V), results of the peritoneal equilibrium test, and the administration of thiamin-containing vitamin supplements were obtained from their medical records.

### Dietary data

The participants were asked how frequently they consumed foods containing antithiamin factors, which included raw fish and shellfish, tea, coffee, and certain vegetables, via a food frequency questionnaire. They were then instructed to complete a 3-day prospective food record to obtain information on daily thiamin, energy, and macronutrient intake. A certified dietitian provided instructions on how to complete the record accurately. The data from the records were verified by the dietitian and analyzed using INMUCAL–Nutrients (version 4.0) software, which is based on the Thai food database (Institute of Nutrition, Mahidol University, Nakhon Pathom, Thailand) [14–16]. The intakes of thiamin, energy, and protein were subsequently compared with the age- and sex-specific recommendations set by the Thai DRI [17]. The dietitian who analyzed the dietary data was blinded to the erythrocyte transketolase activity (ETKA) assay results.

### ETKA assay

The ETKA assay was selected for the laboratory assessment of thiamin status. ETKA was measured at baseline and after the addition of exogenous thiamin pyrophosphate using the method specified in a study by Densupsoontorn et al. [16]. The result, referred to as the thiamin pyrophosphate effect (TPPE), was calculated via the following formula:

$$\%TPPE=\left(\left[ETKA\, activated-ETKA\, basal\right]/ETKA\, basal\right)\times 100$$

A TPPE of 15% to 24.9% indicated TD, and a TPPE of 25% or greater indicated severe TD. The investigators conducting the ETKA assays were blinded to the dietary data and the underlying diseases of the participants.

### Statistical analysis

Continuous data are presented as means and standard deviations if normally distributed and as medians (25^th^ percentile, 75^th^ percentile) if not normally distributed. Categorical data are expressed as counts and percentages. Independent Student’s *t* tests and Mann‒Whitney U tests were used to compare the differences between two continuous variables. The chi-square test was used to determine the associations between two categorical variables.

Linear regression analyses were used to determine the associations between TPPE values and various factors in the CKD group. Initially, simple linear regression of TPPE values on various factors was performed to select potential candidates with a *p* value < 0.1 for inclusion in the subsequent multiple linear regression model, which was conducted using a stepwise procedure. Multicollinearity among independent variables was assessed with a collinearity tolerance threshold of less than 0.1 and a variance inflation factor of greater than 10. Statistical analyses were executed via IBM SPSS Statistics, version 29 (IBM Corp, Armonk, NY, USA), with the significance level set at *p* < 0.05.

## Results

### Demographic and dietary characteristics of CKD and healthy control groups

Thirty-two patients with CKD stage 5D undergoing PD and 34 healthy children were recruited for the study. The anthropometric and dietary data of the participants in the CKD group and the healthy control group are shown in Table 1. The participants in the CKD group did not have wasting or thinness, but 25% of them had stunting. The total thiamin intake per 1,000 kcal of energy, which comprised thiamin from the diet and vitamin supplements, was greater in the CKD group than in the healthy control group (2.14 [1.83, 2.99] vs. 0.87 [0.59, 1.14] mg/1000 kcal; *p* < 0.001). When total thiamin intake was considered, a greater proportion of participants in the CKD group than in the healthy control group had thiamin intake that meets DRI requirements (91% vs. 65%, *p* = 0.012). However, when focusing solely on thiamin intake from dietary sources, a greater proportion of participants in the CKD group than in the healthy control group failed to meet the DRI requirements for thiamin (25% vs. 65%, *p* = 0.001). Additionally, the CKD group had lower percentages of energy and protein intake relative to the DRI recommendations than did the healthy control group (*p* = 0.014 and *p* < 0.001, respectively).

**Table 1:** Characteristics of participants from the chronic kidney disease and healthy control groups^a^

Characteristics	CKD group (*n* = 32)	Healthy control group (*n* = 34)	*p* value
Age (years)^b^	14.7 (10.1, 16.5)	13.9 (1.5, 16.4)	0.228
Male	20 (63)	14 (41)	0.083
BMI-for-age z-score^c^	0.11 ± 1.15	−0.02 ± 0.95	0.369
Wasting or thinness^d^	0 (0)	0 (0)	–
Height-for-age z-score^c^	−1.48 ± 1.17	−0.51 ± 0.79	0.060
Stunting^e^	8 (25)	0 (0)	0.002*
Total thiamin intake per energy (mg/1000 kcal)^b^^,f^	2.14 (1.83, 2.99)	0.87 (0.59, 1.14)	< 0.001*
Dietary thiamin intake per energy (mg/1000 kcal)^b^	0.74 (0.52, 1.00)	0.87 (0.59, 1.14)	0.259
Total thiamin intake (% of DRI)^b,^^f^	160 (133, 253)	117 (71, 202)	0.022*
Dietary thiamin intake (% of DRI)^b^	65 (38, 98)	117 (71, 202)	< 0.001*
Total thiamin intake meeting DRI^g^	29 (91)	22 (65)	0.012*
Dietary thiamin intake meeting DRI^g^	8 (25)	22 (65)	0.001*
Intake of thiamin-fortified milk/formula	4 (13)	11 (32)	0.054
Intake of foods containing antithiamin factors	18 (56)	26 (77)	0.082
Energy intake (% of DRI)^c^	47 ± 19	77 ± 30	0.014*
Protein intake (% of DRI)^b^	69 (53, 117)	127 (80, 209)	< 0.001*
TPPE (%)^c^	9.6 ± 6.0	13.4 ± 8.0	0.202
Thiamin deficiency	4 (13)	10 (29)	0.093

*BMI* body mass index, *CKD* chronic kidney disease, *DRI* dietary reference intake, *TPPE* thiamin pyrophosphate effect, *WHO* World Health Organization

^a^Data are expressed as count (percentage) unless otherwise specified

^b^Data are expressed as median (25^th^ percentile, 75^th^ percentile)

^c^Data are expressed as mean ± SD

^d^Wasting is defined as a WHO weight-for-height z-score of < −2 for children aged 5 years or younger, while thinness is defined as a WHO BMI-for-age z-score of < −2 for children older than 5 years of age

^e^ Stunting is defined as a WHO height-for-age z-score of < −2

^f^ Total thiamin intake includes thiamin intakes from dietary sources and vitamin supplements

^g^DRI references to the age- and sex-specific Thai DRI requirements for thiamin

*p < 0.05

### Proportions of TD in CKD and healthy control groups

In this study, 4 out of 32 participants (13%) in the CKD group had TD, whereas 10 out of 34 participants (29%) in the healthy control group had TD (*p* = 0.093). The mean TPPE value for the CKD group was 9.6% ± 6.0%, whereas that of the healthy control group was 13.4% ± 8.0% (*p* = 0.202). All 4 thiamin-deficient participants in the CKD group received 1 mg of thiamin supplement per day. On the other hand, 64% of the thiamin-sufficient participants with CKD received 1 mg of thiamin supplement per day, 25% received more than 1 mg of thiamin supplement per day, and 11% did not receive any thiamin supplement.

### Disease characteristics of CKD participants

As shown in Table 2, the causes of CKD were primarily glomerular disease (59% of the participants), followed by congenital anomalies of the kidney and urinary tract (31%). Other causes accounted for 9% of the cases. Among the 32 participants in the CKD group, 13 (41%) underwent continuous cyclic PD, 11 (34%) were on continuous ambulatory PD, and 8 (25%) received nightly intermittent PD. The median duration of PD was 5.8 months (2.5, 21.2). Furthermore, the median Kt/V was 2.17 (1.88, 2.84), suggesting adequate PD according to the International Society for Peritoneal Dialysis practice recommendations [18]. The results of the peritoneal equilibration test revealed that 3% were classified as low transporters, 19% as low average transporters, 31% as high average transporters, and 28% as high transporters.

**Table 2:** Clinical characteristics of the chronic kidney disease group^a^

Characteristics	CKD patients (*n* = 32)
Causes of CKD	
CAKUT	10 (31)
GD	19 (59)
Others	3 (9)
Modes of PD	
CAPD	11 (34)
CCPD	13 (41)
NIPD	8 (25)
Duration of PD therapy (months)^b^	5.8 (2.5, 21.2)
24-h PD ultrafiltration (mL)^b^	350 (150, 750)
Kt/V^b^ (*n* = 24)	2.17 (1.88, 2.84)
PET (*n* = 26)	
Low transporter	1 (4)
Low average transporter	6 (23)
High average transporter	10 (38)
High transporter	9 (35)
24-h urine volume (mL)^b^ (*n* = 31)	400 (50, 800)

*CAKUT* congenital anomalies of the kidney and urinary tract, *CAPD* continuous ambulatory peritoneal dialysis, *CCPD* continuous cyclic peritoneal dialysis, *CKD* chronic kidney disease, *GD* glomerular disease, *Kt/V* dialyzer clearance of urea multiplied by dialysis time and divided by volume of distribution of urea, *NIPD* nightly intermittent peritoneal dialysis, *PD* peritoneal dialysis, *PET* peritoneal equilibration test

^a^Data are expressed as count (percentage) unless otherwise specified

^b^Data are expressed as median (25^th^ percentile, 75^th^ percentile)

### Factors associated with TD in CKD participants

The results of simple and multiple linear regression analyses are presented in Table 3. In the initial simple linear regression analysis, five factors with a *p* value < 0.1 (PD duration [in months], high-transport peritoneal membrane, total thiamin intake, Kt/V, and BMI-for-age z-score) were considered potential factors and were selected for inclusion in the multiple linear regression analysis. The intake of antithiamin factors was also analyzed but was not significantly associated with TPPE values (β = −3.37, p = 0.117), and thus was not included in the multivariable model. Multiple linear regression analysis revealed three factors that were independently associated with TPPE values: PD duration (in months) (β = + 0.59, *p* < 0.001), high-transport peritoneal membrane (β = + 0.38, *p* = 0.013), and total thiamin intake (β = −0.35, *p* = 0.022).

**Table 3:** Simple and multiple linear regression analyses of values of the thiamin pyrophosphate effect in relation to various factors among chronic kidney disease participants

Factors	Simple linear regression			Multiple linear regression		
	Regression coefficient (95% CI)	β	*p* value	Regression coefficient (95% CI)	β	*p* value
Duration of PD therapy (months)	0.14 (−0.01 to 0.29)	0.34	0.060	0.23 (0.12 to 0.34)	0.59	< 0.001*
High-transport peritoneal membrane	4.84 (0.51 to 9.17)	0.43	0.030	4.38 (1.03 to 7.74)	0.38	0.013*
Total thiamin intake (mg/1000 kcal of energy)	−1.74 (−3.37 to −0.11)	−0.37	0.037	−1.36 (−2.50 to −0.21)	−0.35	0.022*
Kt/V	2.85 (−0.35 to 6.05)	0.37	0.078			
BMI-for-age z-score	−2.31 (−4.06 to −0.56)	−0.44	0.011			

*BMI* body mass index, *CI* confidence interval, *Kt/V* dialyzer clearance of urea multiplied by dialysis time and divided by volume of distribution of urea, *PD* peritoneal dialysis, *TPPE* thiamin pyrophosphate

*p < 0.05

## Discussion

In this study, TD was observed in 13% of the CKD group and 29% of the healthy control group. Further analysis within the CKD group revealed that greater total thiamin intake was associated with improved thiamin status, as indicated by lower TPPE values. Conversely, a longer PD duration (in months) and a high-transport peritoneal membrane status were linked to poorer thiamin status.

The participants in the CKD group had a significantly greater total thiamin intake than did those in the healthy control group, primarily because of the use of thiamin-containing vitamin supplements. Consequently, the proportion of patients with TD was lower in the CKD group. However, when dietary thiamin intake alone was considered, a greater proportion of CKD participants than healthy controls had inadequate intake. This finding may be attributed to the lower overall energy and protein intakes observed in the CKD group.

Furthermore, this study demonstrated that longer PD duration (in months) and high-transport peritoneal membrane status were linked to poorer thiamin status. Patients classified as high transporters were at greater risk of TD owing to rapid solute transport across the peritoneal membrane, necessitating more frequent PD cycles and increased glucose concentrations in the peritoneal dialysate to achieve efficient PD. Additionally, glucose absorbed from the peritoneal dialysate may contribute to thiamin depletion through increased glucose metabolism, which requires thiamin.

TD in pediatric patients with CKD remains a significant concern, particularly among those undergoing PD. In 1994, Warady et al. [9] reported a notably high proportion of TD among infants with CKD receiving PD, with 2 out of 5 infants (40%) affected, despite receiving a daily thiamin supplement of 0.6 mg that increased their total thiamin intake to 277% of the recommended daily allowance. Conversely, a study by Kriley et al. [19] demonstrated that children with CKD on PD who received thiamin supplementation had better thiamin status than control subjects, consistent with the results of the present study.

Dietary thiamin and caloric intake among pediatric patients with CKD can vary significantly and may fall short of the recommended guidelines. Our findings of inadequate intake in this population are consistent with those of previous studies [4, 20–23]. Pereira et al. [4] reported that 90% of children with CKD stage 5D had inadequate dietary thiamin and caloric intake. Similarly, Kim et al. [20] found that the mean nutrient adequacy ratios for thiamin and energy among children with CKD not receiving kidney replacement therapy were 0.8 ± 0.2 and 0.7 ± 0.2 of the Korean DRI, respectively. Hongsawong et al. [21] reported that energy intake among pediatric patients with CKD stage 5D was only 46% of the DRI.

Don et al. [22] reported a mean thiamin intake of 245% (range: 128–408%) of the recommended dietary intake (RDI) or adequate intake (AI) for age in 12 children with CKD on PD or hemodialysis, most of whom received prescribed nutritional support containing B vitamins. However, when nutritional support was excluded from the calculation, the mean dietary thiamin intake dropped to 82% of the RDI or AI for age. Similarly, Tuokkola et al. [23] found that the median thiamin intake from dietary sources among CKD patients on dialysis was 130% (IQR: 86–153%) of the Nordic Nutrition Recommendations. However, subgroup analysis showed that children who were fed only regular food without renal-specific formula had a thiamin intake of 81% (IQR: 68–133%) of the Nordic Nutrition Recommendations, despite having adequate energy intake.

Several studies showed that children with CKD can achieve sufficient thiamin intake [9, 19, 24, 25]. Kriley et al. [19] and Warady et al. [9] reported dietary thiamin intakes of 93% and 135% of the recommended daily allowance, respectively. Thuc et al. [24] reported thiamin intake of 120% of the DRI, and Lindeback et al. [25] found that the mean thiamin intake among patients with CKD was 205 ± 154% of the RDI, with 72.2% of participants meeting the RDI. Notably, all participants in these studies were supplemented with vitamins [9, 19, 24, 25]. Despite these findings, underlying dietary insufficiencies—especially among those undergoing PD—remain a concern.

This persistent issue of low dietary intake may, in part, be explained by the physiological challenges associated with PD. Pereira et al. [4] proposed two potential explanations for the low caloric intake observed: (i) glucose absorption from the peritoneal dialysate, and (ii) abdominal distension caused by the presence of dialysate fluid.

To date, no studies have identified significant predictive factors associated with TD in children with CKD stage 5D undergoing PD. However, Jankowska et al. [8] assessed thiamin loss through PD in adult patients with CKD and reported that thiamin loss could exceed the amount required by the DRI. They did not identify any associations between the transport characteristics of the peritoneal membrane and thiamin loss. Additionally, no correlation was detected between the effluent glucose concentration and the plasma thiamin level.

On the basis of the results of this study, patients who are undergoing PD may still develop TD despite receiving thiamin supplements. Therefore, laboratory screening for TD in patients with CKD on PD could help identify individuals who may require therapeutic doses of thiamin. Moreover, TD among healthy children should not be overlooked, particularly in those with inadequate dietary thiamin intake and high consumption of foods containing either thiaminase or thiamin antagonists.

This was the first study to compare the proportion of patients with TD among pediatric patients with CKD stage 5D on PD with that of healthy controls. The collection of extensive information on PD and nutritional intake allowed for a thorough analysis of the factors associated with TD in CKD patients. This study also has several notable strengths that lend credibility to its findings. Both the dietitian analyzing the dietary data and the investigators conducting the ETKA assays were blinded to each other’s results, reducing bias and improving the validity of the findings. Additionally, the dietary data were validated via the INMUCAL–Nutrients software (which is based on the Thai food database) and did not depend on participant recall.

However, the study was limited by the lack of data on clinical symptoms and signs of TD in both the CKD and healthy control groups. Inclusion of such clinical findings could have further emphasized the value of dietary screening and biochemical assessment in detecting subclinical TD. Furthermore, the study did not directly measure thiamin levels in the peritoneal effluent; thus, it could not provide concrete evidence of thiamin loss through PD.

## Conclusions

Thirteen percent of pediatric patients with CKD stage 5D undergoing PD were found to have TD, which was associated with longer PD duration (in months) and high-transport peritoneal membrane status, while greater total thiamin intake was linked to improved thiamin status. In line with the recent Clinical Practice Points on vitamin management in children with CKD, which recommend considering biochemical assessment when risk factors for deficiency are present [26], screening for thiamin status should be considered in this population, particularly among those receiving prolonged PD, exhibiting high-transport status, or having inadequate thiamin intake. Furthermore, healthy children are also at risk for TD, and conducting dietary history assessments may help identify those at increased risk.

## Supplementary Information

Below is the link to the electronic supplementary material.

Graphical abstract (PPTX 73.2 KB)

## Data Availability

The data that support the findings of this study are available upon request.

## Abbreviations

*AI*: Adequate intake
*CKD*: Chronic kidney disease
*DRI*: Dietary reference intake
*ETKA*: Erythrocyte transketolase activity
*IQR*: Interquartile range
*PD*: Peritoneal dialysis
*RDI *: Recommended dietary intake
*TD*: Thiamin deficiency
*TPPE*: Thiamin pyrophosphate effect
*WHO*: World Health Organization
