# Supplementary material for: Thiamin deficiency in children with chronic kidney disease on peritoneal dialysis and its association with dialysis duration and transport peritoneal membrane status
Source: Pediatr Nephrol. 2025 Jun 23;41(1):177–84. doi: 10.1007/s00467-025-06847-6 (PMC12686079; doi:10.1007/s00467-025-06847-6)
Supplement: Supplementary file 1 — Graphical abstract (PPTX 73.2 KB) [file 467_2025_6847_MOESM1_ESM.pptx]

## Slide 1
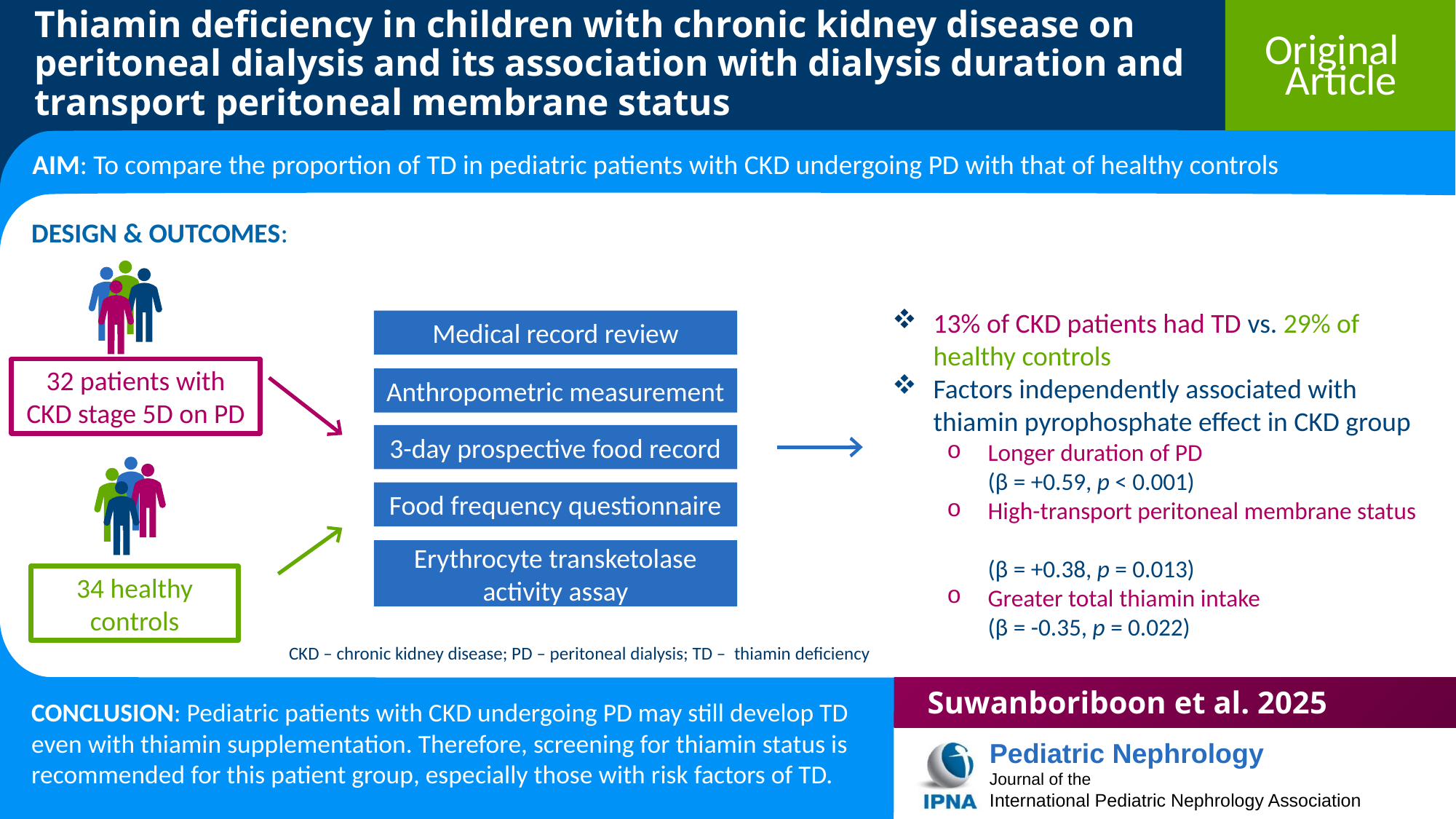

Thiamin deficiency in children with chronic kidney disease on peritoneal dialysis and its association with dialysis duration and transport peritoneal membrane status
AIM: To compare the proportion of TD in pediatric patients with CKD undergoing PD with that of healthy controls
DESIGN & OUTCOMES:
13% of CKD patients had TD vs. 29% of healthy controls
Factors independently associated with thiamin pyrophosphate effect in CKD group
Longer duration of PD
(β = +0.59, p < 0.001)
High-transport peritoneal membrane status
(β = +0.38, p = 0.013)
Greater total thiamin intake
(β = -0.35, p = 0.022)
Medical record review
32 patients with CKD stage 5D on PD
Anthropometric measurement
3-day prospective food record
Food frequency questionnaire
Erythrocyte transketolase activity assay
34 healthy controls
CKD – chronic kidney disease; PD – peritoneal dialysis; TD – thiamin deficiency
Suwanboriboon et al. 2025
CONCLUSION: Pediatric patients with CKD undergoing PD may still develop TD even with thiamin supplementation. Therefore, screening for thiamin status is recommended for this patient group, especially those with risk factors of TD.
